# Controlled ultrasonic interventions through the human skull

**DOI:** 10.3389/fnhum.2024.1412921

**Published:** 2024-06-24

**Authors:** Matthew G. Wilson, Thomas S. Riis, Jan Kubanek

**Affiliations:** Department of Biomedical Engineering, University of Utah, Salt Lake City, UT, United States

**Keywords:** transcranial focused ultrasound, drug release, neuromodulation, skull, correction, compensation, deterministic, intensity

## Abstract

Transcranial focused ultrasound enables precise and non-invasive manipulations of deep brain circuits in humans, promising to provide safe and effective treatments of various neurological and mental health conditions. Ultrasound focused to deep brain targets can be used to modulate neural activity directly or localize the release of psychoactive drugs. However, these applications have been impeded by a key barrier—the human skull, which attenuates ultrasound strongly and unpredictably. To address this issue, we have developed an ultrasound-based approach that directly measures and compensates for the ultrasound attenuation by the skull. No additional skull imaging, simulations, assumptions, or free parameters are necessary; the method measures the attenuation directly by emitting a pulse of ultrasound from an array on one side of the head and measuring with an array on the opposite side. Here, we apply this emerging method to two primary future uses—neuromodulation and local drug release. Specifically, we show that the correction enables effective stimulation of peripheral nerves and effective release of propofol from nanoparticle carriers through an *ex vivo* human skull. Neither application was effective without the correction. Moreover, the effects show the expected dose-response relationship and targeting specificity. This article highlights the need for precise control of ultrasound intensity within the skull and provides a direct and practical approach for addressing this lingering barrier.

## 1 Introduction

One in three patients with a neurological or mental disorder does not respond to drugs or other forms of treatment (Ferguson, 2001; Karceski, 2007; Louis et al., 2010; Al-Harbi, 2012; Jaffe et al., 2019). Neuromodulation provides these treatment-resistant patients with new treatment options, promising to treat brain disorders at their neural sources in deep brain limbic, thalamic, or basal ganglia circuits (Kuhn et al., 2009; Price and Drevets, 2012; Larson, 2014; Widge and Dougherty, 2015; Elias et al., 2016; Dandekar et al., 2018). Unfortunately, current neuromodulation approaches have significant limitations, which make them applicable only to certain indications and patients. In particular, invasive approaches such as deep brain stimulation suffer from a high risk-benefit ratio, which has limited their applications to movement disorders (Larson, 2014). On the other hand, the more flexible, non-invasive neuromodulation approaches currently do not have the necessary spatial specificity in the affected deep brain regions. For instance, electroconvulsive therapy induces brain-wide seizures (Lisanby, 2007), which often lead to cognitive side effects such as memory loss (Ingram et al., 2008). Transcranial magnetic stimulation (TMS) primarily modulates cortical regions of the brain, which limits its ability to act deep in the brain and contributes to variable response (Nicolo et al., 2015). These limitations leave millions of patients inadequately treated.

Low-intensity transcranial focused ultrasound enables neuromodulation that combines non-invasiveness with sharp focus at depth. Ultrasound can be focused through the intact skull and scalp into circumscribed deep brain regions [e.g., about 3–5 mm focal diameter when applied through the human skull (Ghanouni et al., 2015; Harary et al., 2018; Riis et al., 2024a)]. The unique combination of precise focusing and non-invasiveness provides the ability to modulate specific deep brain networks directly, selectively, and flexibly. Two emerging approaches based on low-intensity ultrasound have a high potential for providing effective and safe modulation of deep brain circuits.

One, the mechanical effects associated with the ultrasonic pressure wave can be harnessed to impact drug-loaded nanoparticle carriers (Airan et al., 2017; Wang et al., 2018; Lea-Banks et al., 2021). This way, the drug is released only at the desired ultrasound focus and does not affect other neural circuits or organs. This eliminates the side effects associated with systemic drug administrations. This method has the potential to locally deliver psychoactive drugs that naturally pass through the blood-brain barrier.

Two, the mechanical effects associated with the ultrasonic pressure wave modulate the activity of neurons and ion channels specifically at the ultrasound target, thus providing spatially-specific neuromodulation. These effects modulate neural activity or local connectivity, depending on the stimulus duration (Naor et al., 2016; Kubanek, 2018; Tyler et al., 2018; Blackmore et al., 2019). Brief stimuli, on the order of a second or less, induce transient changes in neural activity (Kubanek et al., 2020; Riis and Kubanek, 2021). For example, brief, 300 ms pulses of ultrasound delivered into specific brain regions induce trial-by-trial changes in choice behavior of non-human primates (Kubanek et al., 2020; Webb et al., 2022). Longer, 30 s exposures led to more sustained effects (Webb et al., 2023) without detrimental consequences. Such sustained effects can also manifest in durable changes in functional connectivity (Fouragnan et al., 2019; Verhagen et al., 2019; Khalighinejad et al., 2020). Mechanistically, ultrasound mechanically activates ion channels (Kubanek et al., 2016, 2018; Prieto et al., 2018; Oh et al., 2019; Yoo et al., 2022). The sustained effects are, at least in part, due to activation of the supporting glial cells (Nanou and Catterall, 2018; Oh et al., 2019).

Nonetheless, both approaches have thus far shown limited effectiveness in large animals and humans. The ultrasound-based targeted drug release from nanoparticle carriers has not yet been performed in large animals or humans. In addition, ultrasonic neuromodulation in humans (Legon et al., 2014; Lee et al., 2016; Ai et al., 2018; Badran et al., 2020; Fomenko et al., 2020) has been much less effective and robust compared with studies in rodents (Tufail et al., 2010; Ye et al., 2016; Lee et al., 2018).

**Figure 1:**
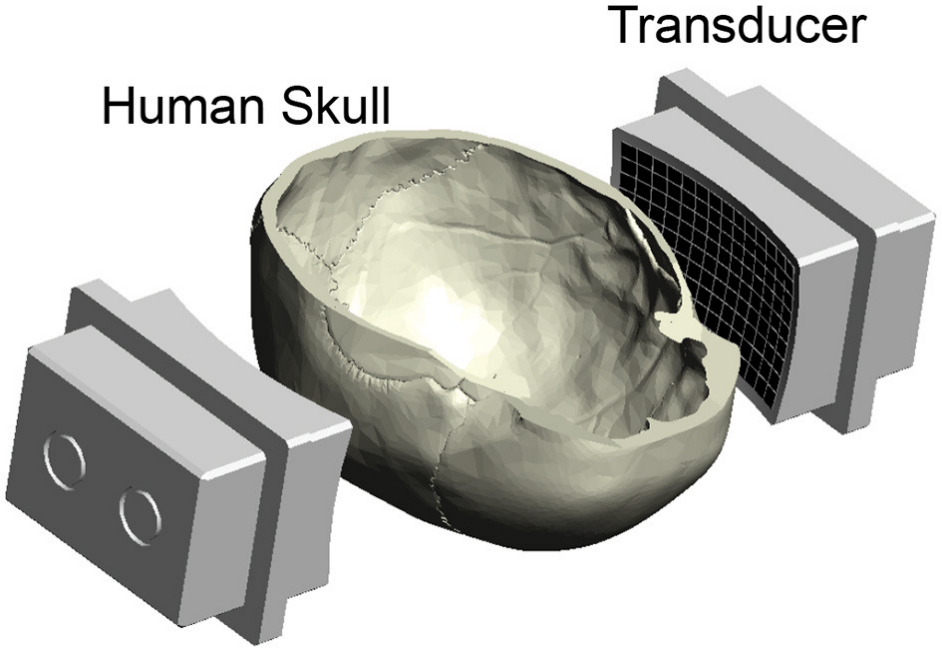
Ultrasound applied through the human skull. Ultrasound was emitted into a central location positioned within a human skull. The ultrasound was delivered using a previously published device (Riis et al., 2023a), consisting of two spherically focused arrays of 126 elements each. They are positioned to transmit through the parietal and temporal bones of the skull (Riis et al., 2023a). The bottom half of the skull was open for insertion of a hydrophone for field characterization, vials of nanoparticle solution for drug release, and fingers for nerve stimulation (Figure 5).

The limitations lie primarily in the thick and acoustically complex human skull (Fry and Barger, 1978; White et al., 2006; Webb et al., 2018; Riis et al., 2021) (Figure 1). The intensity of neuromodulatory ultrasound is attenuated by the skull by a factor of 4.5–64, depending on skull segment and individual (Riis et al., 2021). This large variability in the attenuation factor makes it difficult to provide a confident estimate on the delivered intensity.

To address this issue, we have previously developed a new approach that measures the ultrasound attenuation and dephasing by the head using ultrasound (Riis et al., 2024a). Although the approach has shown promising results through *ex-vivo* skulls (Riis et al., 2024a) and neuromodulation in humans (Riis et al., 2023a,b, 2024a,b), it has not yet compared effectiveness of ultrasound-based drug release and neuromodulation with and without the correction. This study evaluated the applicability of this new approach to drug release and neuromodulation through the human skull. We hypothesize that the correction for the skull (Riis et al., 2024a), if accurate, should lead to drug release and neuromodulation rates that are similar to the case of no skull present.

## 2 Results

### 2.1 Controlled drug release through the human skull

**Figure 2:**
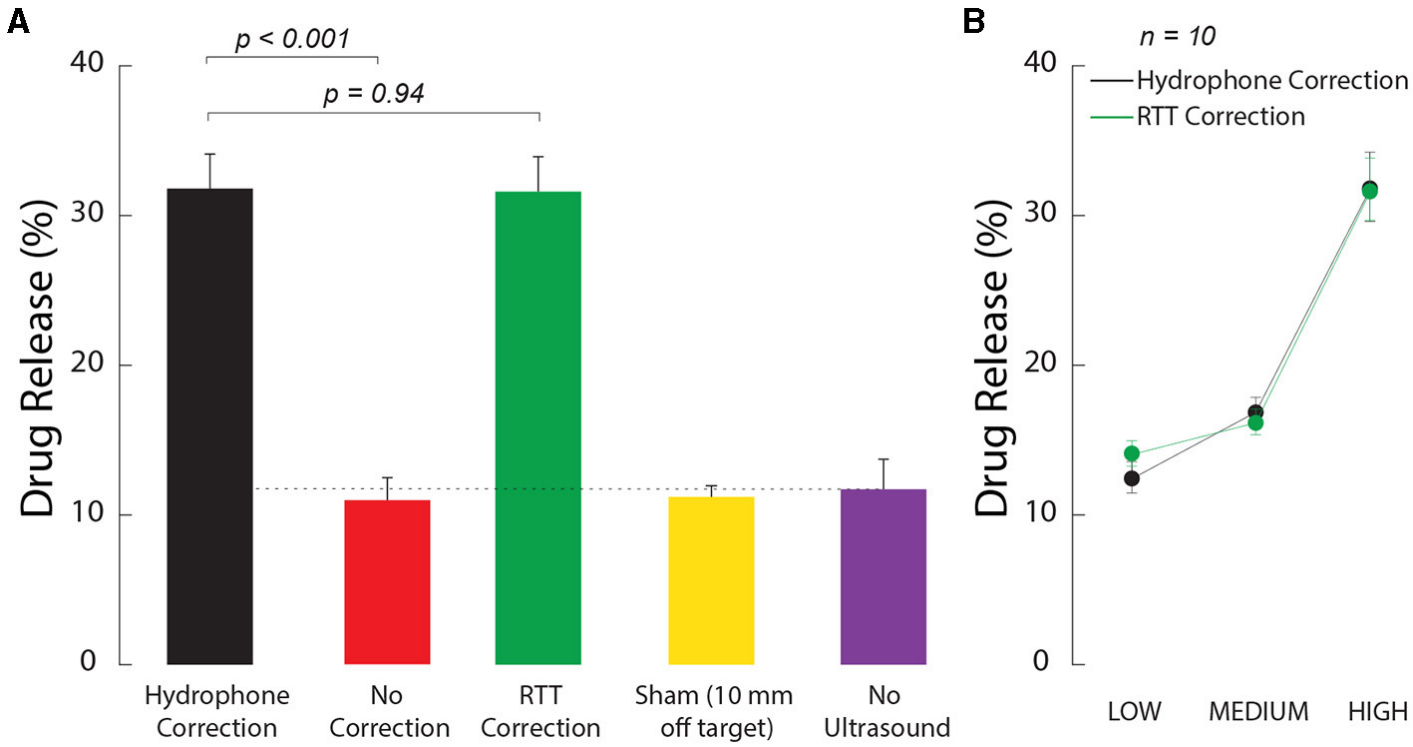
Ultrasound correction for the skull using RTT enables effective and dose-dependent local drug release. **(A)** RTT enables effective drug release from nanoparticle carriers. Safe, biocompatible nanoparticle carriers (Wilson et al., 2024) encapsulated the neuromodulatory drug propofol. The nanoparticles release their drug load when impacted by low to medium intensity ultrasound. Vials with the nanoparticles were positioned within a central location of an *ex-vivo* skull. Ultrasound, delivered through the skull, impacted the nanoparticles in 100 ms pulses delivered every 1 s for 60 s, at a frequency of 650 kHz and pressure amplitude of 1.8 MPa. The data were collected under the hypothetical ideal correction for the skull (black), without any correction (red), and after applying RTT (green). A sham condition delivered the stimulus 10 mm below the vial (yellow) after using the hydrophone correction for the skull. A second sham condition placed the vial at target but delivered no ultrasound (purple). The dotted line represents the baseline when no ultrasound is applied. The baseline can be non-zero due to free (unencapsulated) drug or due to the nanoparticles being partially leaky (Wilson et al., 2024). The individual conditions were randomly interleaved. The bars comprise *n* = 10 distinct samples, with the exception of the No Ultrasound case, which used *n* = 6. The error bars represent the s.e.m. **(B)** Dose-response relationship. The LOW, MEDIUM, and HIGH labels correspond to an intended peak pressure of 1.2, 1.5, and 1.8 MPa, as measured in free-field. All datapoints comprised *n* = 10 distinct samples. The error bars represent the s.e.m.

First, we tested whether the ultrasound-based method, referred to as relative through-transmit [RTT, (Riis et al., 2024a)], could be used to release drugs at clinically-relevant and deterministic doses in deep brain targets. To do so, we prepared ultrasound-sensitive nanoparticle carriers as in previous studies (Rapoport, 2016; Airan et al., 2017; Wilson et al., 2024), and encapsulated in the nanoparticles the neuromodulatory drug propofol. We then tested how the nanoparticles respond to ultrasound when RTT correction is applied and when it is not. We found that RTT is critical to mediate effective release when ultrasound is applied through the skull (Figure 2A). Without RTT (red), the amount of detected drug was no different from the no stimulation case (purple, *t*_14_ = 0.30, *p* = 0.77, two-sample two-tailed *t*-test). The application of RTT (green) nearly tripled the release effectiveness (factor of 2.9 increase), releasing 31.6% of the encapsulated propofol. This level was not statistically different (*t*_18_ = 0.08, *p* = 0.94, paired two-tailed *t*-test) from the 31.8% release obtained with the hypothetical best-possible correction (black).

We confirmed the spatial specificity of the release using an active sham condition in which ultrasound was delivered with the ideal hydrophone correction 10 mm below each vial (Figure 2A, yellow bar). In this case, the amount of detected drug was no different (*t*_14_ = 0.30, *p* = 0.77) from the case in which no ultrasound was applied (purple).

We investigated the dose dependence of the release. Specifically, we varied the stimulation across three intensity levels, all within the FDA 510 (k) Track 3 guidelines (FDA, 2019). We found an increase in stimulation effectiveness with increasing level of the ultrasound (Figure 2B). The effect of the stimulation level was highly significant (two-way ANOVA, *F*_2, 54_ = 84.53, *p* = 2.3 × 10^−17^). The release levels were statistically indistinguishable from the hypothetical ideal correction (green vs. black; two-way ANOVA, *F*_1, 54_ = 0.02, *p* = 0.89), and there was no significant interaction between the two factors (*F*_2, 54_ = 0.35, *p* = 0.7).

### 2.2 Controlled nerve stimulation through the human skull

**Figure 3:**
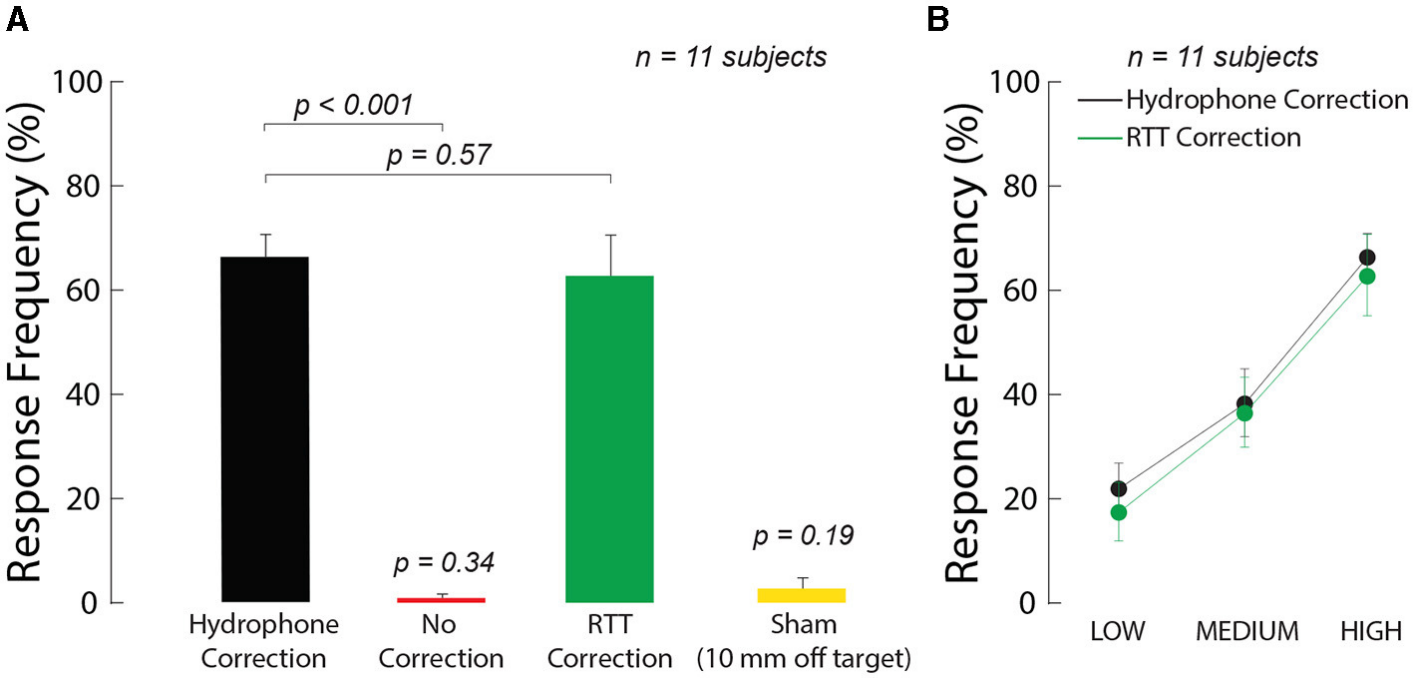
Ultrasound correction for the skull using RTT enables effective ultrasonic stimulation through the skull. **(A)** RTT enables effective modulation of peripheral nerves through skull. The arrays targeted nerves in the thumb of 11 participants. The thumb was secured in the central target inside an *ex-vivo* skull. The arrays delivered into the target a 300 ms stimulus at a frequency of 650 kHz and pressure amplitude of 1.8 MPa. The data were collected with the ideal correction (black), without any correction (red), and after applying RTT (green). A sham condition delivered the stimulus, with hydrophone correction, 10 mm below the finger (yellow). The individual conditions were presented randomly every 8–12 s, for a total of 10 repetitions. Subjects reported any nociceptive response, which indicates stimulation of nerves and nerve endings. Response frequency represents the proportion of trials in which subjects reported a nociceptive response. **(B)** Dose-response relationship of the stimulation. There was a significant modulation by the ultrasound pressure but no significant difference in the responses following the ideal (black) and RTT (green) corrections (see text for details). The LOW, MEDIUM, and HIGH labels correspond to an intended peak pressure of 1.3, 1.55, and 1.8 MPa, as measured in free-field. The error bars represent the s.e.m.

We further tested the ability of the method to provide effective neuromodulation. We specifically focused on responses of peripheral nerves within the human thumb, which we have characterized in free field (Riis and Kubanek, 2021). This preparation carries three key advantages (Riis and Kubanek, 2021): (1) it features intact human nerves (2) there is no confound of anesthesia, and (3) there is no stimulation artifact since responses are assessed at the behavioral level. Specifically, we instructed 11 human subjects to place their thumb into a holder at the central target inside an *ex-vivo* skull. We quantified the subjects' responsiveness to the ultrasound when RTT was applied and when it was absent. We delivered into the target a 300 ms stimulus of specific pressure levels and assessed the effects on the subjects' nociceptive responses. Nociceptive responses indicate stimulation of nerves or nerve endings in the tissue (Gavrilov et al., 1977; Gavrilov, 2016; Riis and Kubanek, 2021). We found that RTT was critical for effective stimulation (Figure 3A). Without RTT, there was no significant stimulation (red; *t*_11_ = 1.00, *p* = 0.34, one-sample two-tailed *t*-test). Following RTT, the response rate of subjects to the stimuli reached 62.7%. This level was not statistically different (*t*_10_ = 0.58, *p* = 0.57, paired two-tailed *t*-test) from a 66.3% response rate obtained with the hypothetical best-possible ground-truth correction, which is as good as if no skull was present. To control for potential confounds that could be associated with ultrasonic stimulation, we randomly interleaved a sham stimulus with the ideal hydrophone correction for the skull that delivered the ultrasound 10 mm below the target. This off-target stimulation produced no significant stimulation (yellow, *p* = 0.19, one-sample two-tailed *t*-test, *t*_11_ = 1.39). This controls for a potential artifactual effect and confirms the spatial specificity of the stimulation.

**Figure 4:**
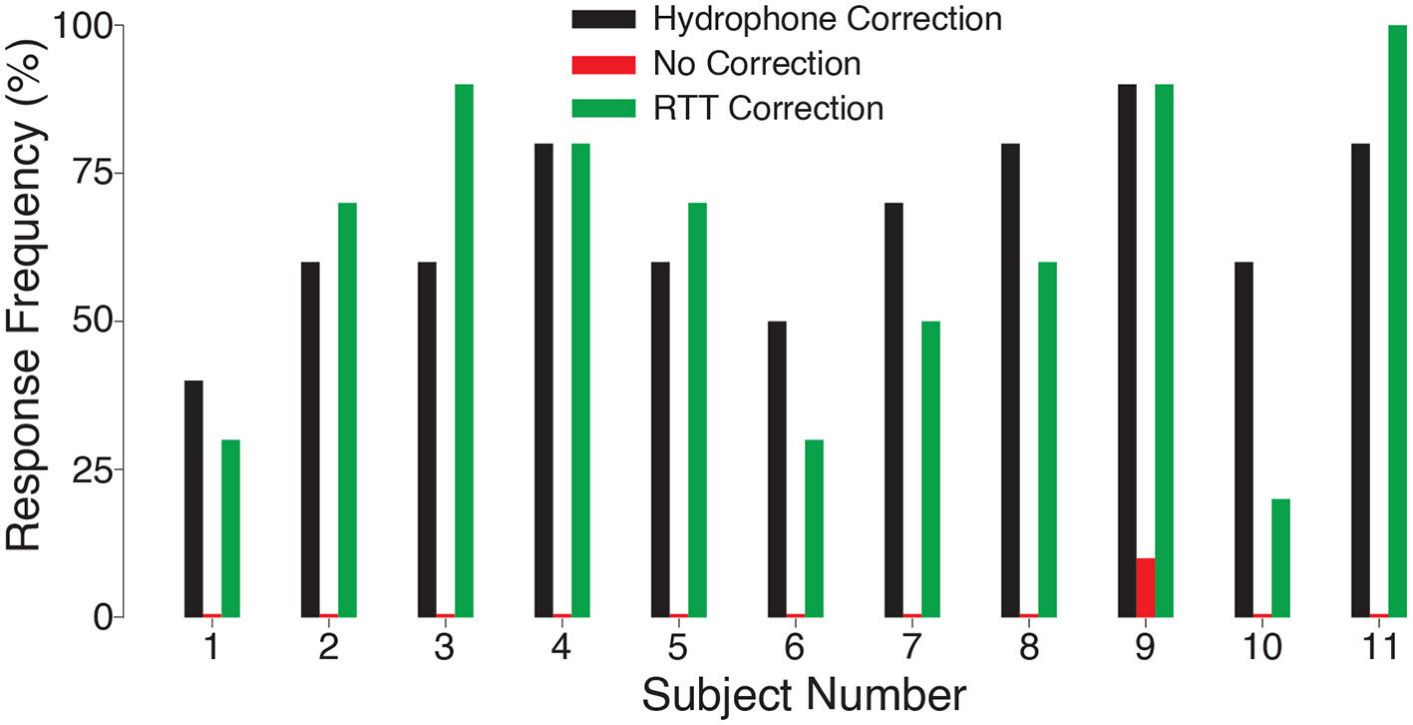
Nerve stimulation in each subject. Response rates for the ideal (black) and RTT (green) correction for each individual subject. When no correction was applied, there was no significant ultrasonic nerve stimulation (red; *t*_11_ = 1.00, *p* = 0.34, one-sample t-test) and only subject 9 responded.

To investigate the effect of intensity on the stimulation effect, we varied the delivered ultrasound intensity across the same levels as in Figure 2. We found an increase in stimulation effectiveness with increasing level of the ultrasound (Figure 3B). The response frequency reached 62.7% for the strongest (1.8 MPa) stimulus, and was significant also for the weakest stimulus tested (1.3 MPa; *t*_10_ = 7.63, *p* = 1.7 × 10^−5^, one-sample two-tailed *t*-test). The effect of the stimulation level was highly significant (two-way ANOVA, *F*_2, 60_ = 25.24, *p* = 1.1 × 10^−8^). The responses were statistically indistinguishable from the hypothetical ideal correction (green vs. black; two-way ANOVA, *F*_1, 60_ = 0.41, *p* = 0.52), and there was no significant interaction between the two factors (*F*_2, 60_ = 0.20, *p* = 0.98). We further evaluated the data separately for each subject (Figure 4). The figure leads to the same general conclusions, suggesting that the results were not due to an averaging artifact. There were no detrimental effects reported by the subjects during or following the stimulation. Together, these data (1) showed that the ultrasound correction for the head using RTT is critical for effective transcranial stimulation of nerves, (2) suggested a range of ultrasound pressures necessary for nerve stimulation, and (3) validated the biological safety of the stimulation.

## 3 Discussion

In this study, we have demonstrated the major impact of the skull on the effectiveness of ultrasound-based inteventions. We have developed a direct and practical method that measures and compensates for the skull. The compensation restores the desired drug release and neuromodulation effects at the ultrasound target. The method is practical in that it is performed in seconds, and does not require skull scans, simulations, or parameters.

Specifically, with no correction for the skull, drug release from ultrasound-responsive nanoparticles was no different from the release without ultrasound (*t*_14_ = 0.30, *p* = 0.77) and stimulation of nerves in fingers elicited no significant response subjects (*t*_11_ = 1.00, *p* = 0.34). In contrast, the relative through transmit approach was able to restore rates of drug release and finger stimulation with no detectable difference from a perfect correction obtained from hydrophone measurements (*F*_1, 54_ = 0.02, *p* = 0.89; *F*_1, 60_ = 0.41, *p* = 0.52; respectively).

While ultrasound-triggered drug release has been demonstrated in rodents (Airan et al., 2017; Zhong et al., 2019; Lea-Banks et al., 2020; Lea-Banks and Hynynen, 2021), its effectiveness in humans remains to be shown. The skull has been a major barrier to a successful translation of this approach. We have demonstrated here a method that leads to effective drug release inside a central target positioned within the human skull. We have shown that this or related correction for the skull will be critical for clinical translation to enable precise dose control.

Depending on the drug encapsulated in the nanoparticles, this approach may be applied for a variety of applications. Releasing propofol, as described here, could be useful as a tool to probe the function or diagnose dysfunction in the brain by transiently suppressing specific areas. Indeed, this approach has been used to suppress seizure activity in rodents (Airan et al., 2017). A similar approach in humans would be useful for causally probing the origins of epileptic seizures before more lasting intervention. Pharmacological treatments of mental disorders may also benefit from localized drug release; for instance, depression treatments are frequently limited by the prevalence of unwanted side effects. Recent approaches to treat depression with neuroplasticity-inducing drugs such as ketamine or other psychedelics have been limited in scope due to their psychoactive effects (Vargas et al., 2021). Delivering such drugs to specific parts of the brain involved in an individual's condition could allow for selective rewiring and related neuroplastic effects in those areas. Another natural application would be for chemotherapy of brain tumors such as glioblastoma. This diagnosis has high mortality (Smoll et al., 2013), and drugs have limited effectiveness at doses which can be tolerated by patients. Localized release of these drugs, potentially in tandem with blood brain barrier disruption (Idbaih et al., 2019; Gould et al., 2023), could improve the standard of care for these patients.

Transcranial focused ultrasound has tremendous potential to non-invasively modulate neural activity in the brain. Its effects have been attributed to a variety of mechanisms such as direct ion channel activation, effects on cell membranes, or heating of tissue (Kubanek et al., 2016; Plaksin et al., 2016; Darrow et al., 2019; Darmani et al., 2022). It is now well established that effective neuromodulation requires the delivery of a tightly controlled, predictable ultrasound intensity at target. Moreover, the intensity also dictates whether the net effect is excitatory or inhibitory (Plaksin et al., 2016). Here, we have demonstrated that the RTT technique can effectively stimulate nerves through the human skull. The correction was robust such that the response rates could not be statistically distinguished from the response rates obtained using a calibrated hydrophone (Figure 3). Together, the skull correction approach maximizes effectiveness while not risking an intensity excess and potential harm; which could occur if operators did not have a tool to deliver into the brain controlled, deterministic level of ultrasound intensity.

Besides therapies, ultrasonic neuromodulation has the potential to serve a guidance tool. For instance, deep brain stimulation (DBS), which implants electrodes into tissues, has shown promise for treatments of a variety of mental and neurological disorders (Drobisz and Damborská, 2019). However, due to the invasive nature of DBS, probing multiple potential treatment sites has not been feasible. Ultrasonic neuromodulation is non-invasive and flexible and can thus causally probe these areas to determine effective implant sites for each individual patient. Using the skull correction method demonstrated here, clinicians are now empowered to modulate these areas using a predictable ultrasound intensity.

This article has certain limitations. First, to accurately control the dose of a drug released at the target, it will be necessary to quantify the amount of nanodroplets remaining in the blood stream in addition to the amount of drug released from nanoparticles—a factor which is not accounted for here. Such an approach may utilize the enhanced echogenicity of perfluorocarbon-based drug carriers (Rapoport et al., 2011) or acoustic emissions from drug carriers undergoing ultrasound stimulation (Lea-Banks et al., 2020). The model used here is also not necessarily an accurate representation of nanoparticles in the bloodstream as many other factors are present in the blood and the mechanical environment is substantially different. However, while the model does not inform on absolute quantities of the released drugs, it did validate the need for and accuracy of the key compensation for the head, and demonstrated a clinically useful application. Second, neuromodulation likely depends on which structures are stimulated, and the excitable structures stimulated here may not be representative of neurons in the brain. Nonetheless, again, our goal here was not to find a perfect neuromodulation protocol; instead we used the excitable tissue to validate the need for and accuracy of the ultrasound-based skull correction method, RTT. Finally, we have only evaluated these applications with one skull. To address this shortcoming, we have previously demonstrated the functionality of RTT to restore ultrasound intensity through eight skulls (Riis et al., 2024a), though that study did not apply the method to actual interventions.

This work highlights the need to compensate for the human skull and provides evidence for the feasibility of effective ultrasound-based interventions through the human skull. We have presented a practical, imaging- and parameter-free method that measures and compensates for the skull, thus providing intended level of drug release and intended magnitude of neuromodulation. Addressing this key barrier provides the critical step toward using transcranial ultrasound for precision, circuit-oriented treatments of mental and neurological disorders.

## 4 Materials and methods

### 4.1 Ultrasonic hardware

The ultrasound hardware system used two spherically focused arrays described in detail in previous publications (Riis et al., 2023a, 2024a). The arrays had a radius of 165 mm, 126 elements, 9 × 14 element grid, with inter-element spacing of 0.5 mm. Each array had a height of 55 mm and a width of 86 mm. They were mounted to a rigid plastic frame positioned opposite each other and separated by a distance of 180 mm. This allows the ultrasound to be delivered through the parietal and temporal bones of *ex-vivo* skulls. The transducers were driven by a programmable system (Vantage256, Verasonics) with an external power supply (QPX600DP, Aim and Thurlby Thandar Instruments). A schematic of the ultrasound transducers with the skull is shown in Figure 1.

### 4.2 Skulls

One *ex-vivo* human skull was used in this study. The skull was obtained from Skulls Unlimited (Oklahoma City, OK). The supplier provides *ex-vivo* specimens specifically for research under a research agreement. A large opening was made at the bottom of the skull to enable field measurements inside the skull. Each skull was degassed overnight in deionized water (Fry and Barger, 1978). Following the degassing at −25 mmHg, the skull was transferred, within the degassed water, into an experimental tank filled with continuously degassed water (AIMS III system with AQUAS-10 Water Conditioner, Onda).

### 4.3 Targeting and skull correction methods

**Figure 5:**
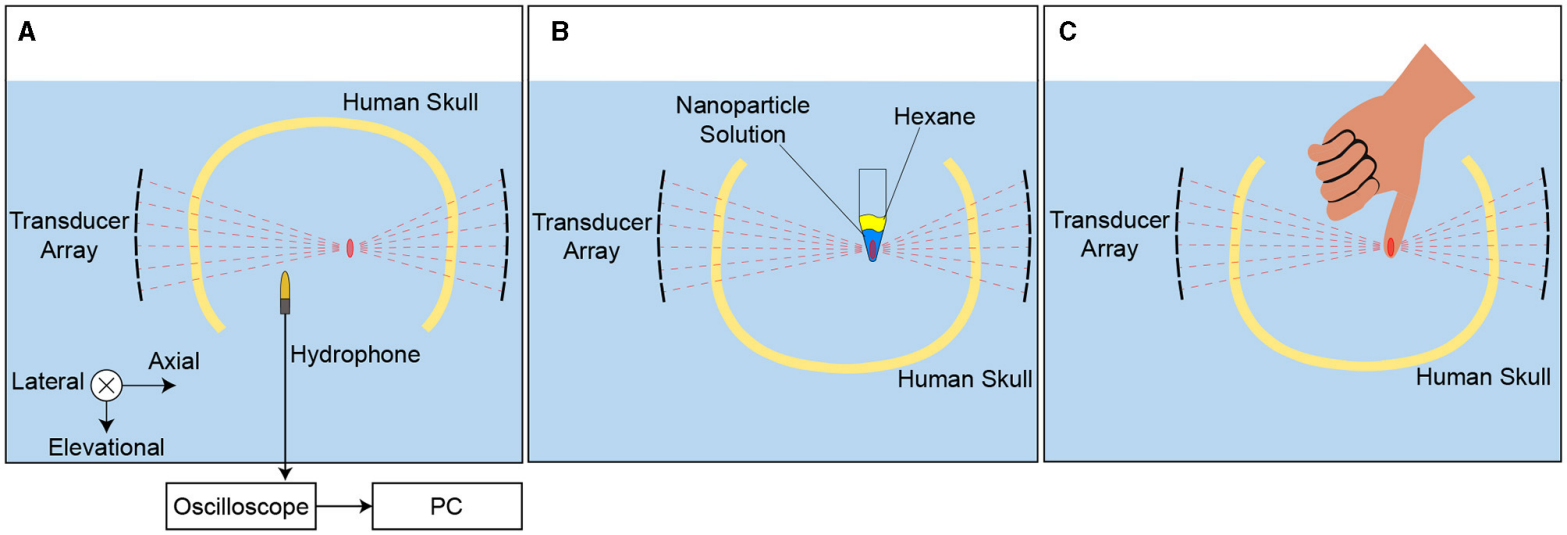
Experimental setup. **A** Hydrophone field scans in a degassed water tank were used for targeting and the ideal hydrophone correction. **B** A 1.5 mL microcentrifuge tube containing propofol-loaded nanoparticles was placed at the ultrasonic focus to assess drug release with different skull correction methods. Drug release into the hydrophobic sink, hexane, placed on top of the nanoparticle solution was measured after sonication. The tubes were mounted in the same location repeatedly with a custom 3-D printed part. **C** Subjects placed a thumb at the ultrasonic focus to assess nerve stimulation with different correction methods. The subjects responded verbally any time they perceived the ultrasound stimulation. A custom 3-D printed positioner was used to guide finger placement.

The targeting and skull correction methods used here are the same as described previously (Riis et al., 2024a) and described briefly here. Targeting for all correction methods began by mapping the acoustic fields from each element of the transducer using a capsule hydrophone (HGL-0200, Onda) mounted to a 3-degree-of-freedom translation system (Aims III, Onda). To obtain these maps, each element was driven separately with 10 cycles of a 650 kHz, 15 V sine wave. The hydrophone was calibrated for angles ranging from 0 to 90 degrees, and the calibration values at 90 degrees were applied to obtain absolute intensity for our setup. The measurements were performed with and without the skull present. The phase and amplitude correction for each element were computed based on the correction method selected. The ultrasound setup used for field scans is shown in Figure 5A.

Three methods of skull correction were compared in this study: the RTT correction, a hydrophone-based correction, and no correction. With no correction, the free-field (no skull) measurements were used. The hydrophone correction represents a ground-truth correction and is calculated by comparing hydrophone measurements with and without the skull. Specifically, the hydrophone is used to measure the relative speedup and the relative reduction in amplitude due to the presence of the skull. Finally, our RTT correction was calculated by measuring the response from each element of one side of the transducer from each element on the opposing transducer. This enables a direct measurement of phase shift and attenuation caused by the skull for each element. Details and schematics regarding this method can be found in a previous publication (Riis et al., 2024a).

### 4.4 Local drug release

We manufactured ultrasound-sensitive nanoparticles according to previous approaches (Rapoport, 2016; Airan et al., 2017; Wang et al., 2018). The procedure followed that of Wilson et al. (2024), using a perfluorooctyl bromide core (Tokyo Chemical Industry Co., Japan) and encapsulating the neuromodulatory drug propofol (Millipore Sigma, Canada) (Airan et al., 2017; Wang et al., 2018). The nanoparticles were stabilized with a polymeric shell composed of methoxy- poly(ethylene glycol)-b-poly(D,L-lactide) (PEG-PDLLA) co-polymers with 2,000 : 2,200 g/mol molecular weights (PolySciTech, USA). The drug was encapsulated at a concentration of 62.9 μg/mL and a total mass of 12.6 μg.

Freshly prepared nanoparticle emulsions were introduced into vials of 8 mm in diameter (1.5 ml polypropylene microcentrifuge tubes, Globe Scientific) at a volume of 0.2 ml, reaching a height of 7 mm. The acoustic impedance of polypropylene (2.4 MRayl) is close to that of water (1.5 MRayl), resulting in only about 5.3% of the incident energy being reflected (Curry et al., 1990; Meimani et al., 2017). For each ultrasound-release condition (*n* = 10 vials), a vial was placed into a central location of an *ex-vivo* skull, as shown in Figure 5B. A 3D-printed positioner held the vial in place. For hydrophone- and RTT-corrected stimuli, we varied the intended peak pressure levels across 1.3, 1.55, and 1.8 MPa. The ultrasound was pulsed for 100 ms every 1 s for 60 s to align with previous studies (Airan et al., 2017; Wilson et al., 2024). The results shown in Figure 2A were only at the highest pressure level: 1.8 MPa in free-field, 1.84 MPa with hydrophone correction, 0.73 MPa with no correction, and 1.95 MPa with the RTT correction. Propofol released from the nanoparticle emulsions was extracted into a 0.1 mL layer of hexane, as in previous studies (Zhong et al., 2019; Wilson et al., 2024). The concentration of propofol encapsulated then extracted was quantified using UV-Vis spectrophotometry (Nanodrop 2000, Thermo Scientific). We included an additional condition in which no ultrasound was applied to obtain a baseline for diffusion of propofol into hexane.

### 4.5 Nerve stimulation

Eleven subjects participated in the stimulation (three females, eight males, aged between 21 and 39 years). The study was approved by the Institutional Review Board of the University of Utah (Protocol #00130036). All subjects provided informed consent. No subject was excluded. Subjects placed their thumb into the central location of an *ex-vivo* skull as shown in Figure 5C. A 3D-printed positioner held the subjects' thumb in place. The positioner consisted of a rectangular slot 24 mm wide with a 3 mm semicircular groove that constrained the thumbs lateral, axial, and elevational movement to within 3 mm in each direction. The stimulation was performed inside an ultrasound tank filled with continuously degassed water (AIMS III system with AQUAS-10 Water Conditioner, Onda). Since the ultrasound driving electronics emitted sound and light when stimulating, subjects wore noise-canceling earmuffs (X4A, 3M; noise reduction rating of 27 dB) and had their eyes closed. Subjects could not hear or see a scheduled stimulus.

Each subject experienced eight distinct stimuli, presented randomly. The ultrasound stimuli were 300 ms in duration. There were three different correction methods—hydrophone correction, no correction, and RTT correction (see above)—and a sham condition. In the sham condition, which was specifically presented for the ideal, hydrophone correction, the ultrasound was programmatically steered 10 mm below the target. For hydrophone- and RTT-corrected stimuli, we varied the intended peak pressure levels across 1.3, 1.55, and 1.8 MPa. The results shown in Figure 3A were only at the highest pressure level: 1.8 MPa in free-field, 1.84 MPa with hydrophone correction, 0.73 MPa with no correction, and 1.95 MPa with the RTT correction. We performed 10 repetitions of each stimuli producing a total of 80 trials per subject. The stimuli were delivered every 8–12 s and randomized so that subjects could not anticipate their onset or type. The subjects were instructed to report a percept with a verbal command (Pain, Vibration, or Tap). A blinded experimenter recorded the percept associated with each stimulus. The response frequency was computed as the proportion of trials in which a nociceptive response was registered.

## Data availability statement

The raw data supporting the conclusions of this article will be made available by the authors, without undue reservation.

## Ethics statement

The studies involving humans were approved by University of Utah Institutional Review Board. The studies were conducted in accordance with the local legislation and institutional requirements. The participants provided their written informed consent to participate in this study.

## Author contributions

MW: Conceptualization, Data curation, Formal analysis, Investigation, Methodology, Software, Validation, Visualization, Writing – original draft, Writing – review & editing. TR: Conceptualization, Data curation, Formal analysis, Investigation, Methodology, Software, Validation, Visualization, Writing – original draft, Writing – review & editing. JK: Conceptualization, Funding acquisition, Resources, Supervision, Visualization, Writing – review & editing.
